# In-Hospital Outcomes and Long-Term Follow-Up after Percutaneous Transcatheter Closure of Postinfarction Ventricular Septal Defects

**DOI:** 10.1155/2017/7971027

**Published:** 2017-05-16

**Authors:** Ruoxi Zhang, Yong Sun, Meng Sun, Hui Zhang, Jingbo Hou, Bo Yu

**Affiliations:** Department of Cardiology, Key Laboratories of Education Ministry for Myocardial Ischemia Mechanism and Treatment, 2nd Affiliated Hospital of Harbin Medical University, Harbin 150086, China

## Abstract

Postinfarction ventricular septal defects (VSD) represent a devastating complication of acute myocardial infarction and are associated with high mortality. Percutaneous interventional closure of postinfarction VSD has been proposed as a potential alternative to surgery. The study aimed to evaluate the therapeutic safety and efficacy of percutaneous interventional closure of postinfarction ventricular septal defects (VSD). Each patient was assigned to one of two groups, based on whether they died during hospitalization (death group) or survived (survival group) in this retrospective study. In-hospital and follow-up data were analyzed. Placement of the VSD occluder was successful in 12 procedures (80%). The mean defect size was 14.20 ± 4.89 mm. Compared to the patients who died, those who survived had higher systolic blood pressure, diastolic blood pressure, and left ventricular ejection fraction upon admission, as well as lower pulmonary/systemic flow ratio and shorter time from acute myocardial infarction to procedure. The incidence of cardiac shock and class IV heart failure was lower in the survival group than in the death group, and these factors correlated with in-hospital and 30-day mortality. Percutaneous closure of postinfarction VSD is an effective technique, which can be performed with a high procedural success rate.

## 1. Introduction

Postinfarction ventricular septal defect (VSD) is a devastating complication of acute myocardial infarction (AMI) and is associated with high mortality [1]. Moreover, medically managed patients with postinfarction VSD have 30-day mortality rates as high as 94% [2]. In the past, surgical closure was the treatment of choice for this serious complication. However, mortality rates after surgical closure remain high (20–87%) even in current series, with higher rates for patients with advanced age, comorbidities, severe coronary artery disease, or hemodynamic instability [3–6].

It was suggested that percutaneous interventional closure of the ventricular septum may serve as a potential alternative to surgical closure, with the advantages of being less invasive, causing little tissue damage, and promoting immediate VSD closure [7]. However, little is known about the outcomes and long-term follow-up of percutaneous interventional closure of postinfarction VSD. Therefore, we sought to evaluate the therapeutic safety and efficacy of the percutaneous interventional approach performed at our institution.

## 2. Methods

### 2.1. Ethics Statement

The present study was approved by the Research Ethics Committee of the Second Affiliated Hospital of Harbin Medical University, China. Data analysis was blinded to the patients' identification information; therefore, no informed consent was required.

### 2.2. Patients and Study Design

Between August 2003 and February 2015, 15 patients with AMI complicated with VSD were admitted to the cardiology intensive care unit (CCU) of our institution and underwent percutaneous interventional VSD closure in this retrospective study. Inclusion criteria were the presence of VSD as a result of AMI, patient's haemodynamic stabilization. Where technically feasible, device closure was considered as the preferred treatment option without prior attempt at surgical closure. Exclusion criteria were based on echocardiographic findings as follows: large VSD (>35 mm), apical VSD without a suitable rim, or basal VSD located too close to the mitral, tricuspid, or aortic valvular apparatuses; patients could not lie down under the cardiac function at that time.

Each patient was assigned to one of two groups, based on whether they died during hospitalization (death group,* n* = 4) or survived (survival group,* n* = 11). All interventional closure procedures and percutaneous coronary interventions were performed in the Department of Cardiology of our hospital, which is a single, high volume, tertiary interventional treatment center. All procedures were performed by experienced interventional cardiologists with no involvement in the present study. The baseline demographic and angiographic characteristics, the complications, and the laboratory and physical examination data generated during hospitalization were recorded based on a systematic review of the patients' hospital files.

### 2.3. Coronary Angiography and Stenting

Coronary angiography was performed using the percutaneous radial artery approach; the femoral approach was used when an intra-aortic balloon pump was required. All angiographic data obtained from the catheterization laboratory records were assessed using conventional protocols. The target artery was defined to be clinically significant when vessel stenosis was >50%. Blood flow in the infarct-related artery was graded based on the thrombolysis in myocardial infarction (TIMI) trial [8].

A chewable loading dose of 300 mg aspirin and 600 mg clopidogrel with heparin (100 IU/kg) was provided if stenting of coronary arteries was performed. The success of the procedure was defined as achieving <20% stenosis of the infarct-related artery, with TIMI III flow after stenting. All patients received standardized treatment for ST-segment elevation myocardial infarction, which consisted of 100 mg aspirin, angiotensin-converting enzyme inhibitor/angiotensin receptor blocker, 20 mg atorvastatin, 75 mg clopidogrel once a day, and subcutaneous low-molecular-weight heparin twice a day after stenting. Tirofiban was provided when deemed necessary by the interventional cardiologist.

### 2.4. Interventional Closure Procedure

**Figure 1:**
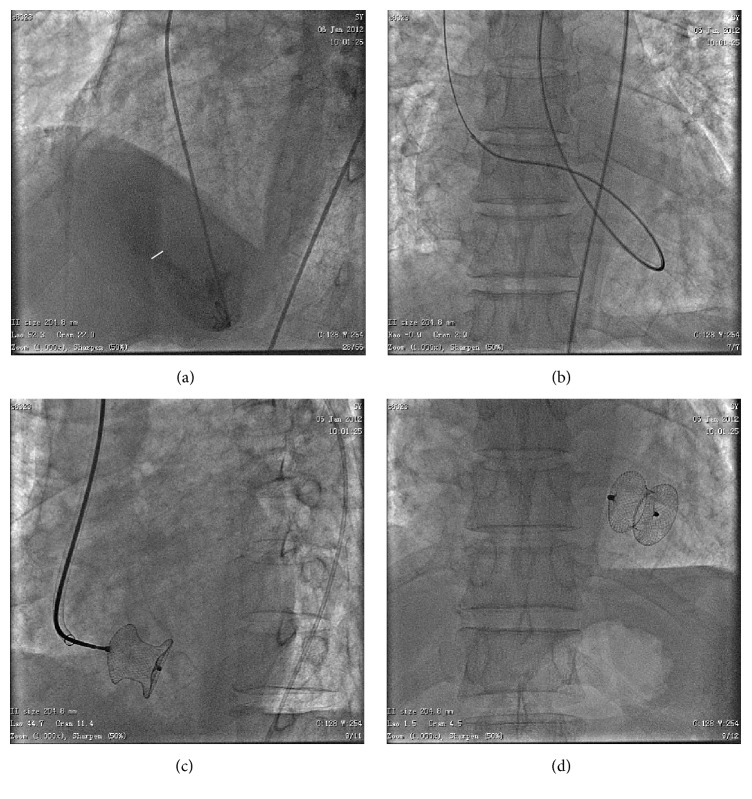
Posteroanterior projection of fluoroscopy images of percutaneous postinfarction ventricular septal defect (VSD) closure. (a) Right ventriculogram showed contrast passing to the left ventricle through an apical VSD. (b) The guidewire was advanced into the pulmonary artery or the superior vena cava, captured using a snare, and exteriorized at the right internal jugular vein for establishing an arterial-venous circuit. (c) The left ventricular disc of a 24 mm muscular VSD device (AGA Medical Corporation, Plymouth, MN, USA) deployed through a 12 F shuttle sheath. (d) Amplatzer muscular VSD occluder.

The procedure was performed under fluoroscopic and echocardiographic guidance. All patients received 100 mg aspirin once a day and heparin (100 U/kg) intravenously. The standard technique of transcatheter VSD closure was followed [9, 10]. First, the right femoral artery was punctured, and a 6–8 French sheath was inserted. Left ventricular angiogram was performed to establish landmarks and determine the location and size of the VSD (Figure 1(a)). A right internal jugular sheath was then inserted, and heparin was administered intravenously. Subsequently, a diagnostic right Judkins catheter was advanced into the left ventricle (LV) and manipulated into the mouth of the VSD, allowing a soft, long guidewire to be passed through the VSD into the right ventricle (RV). The guidewire was advanced into the pulmonary artery or the superior vena cava, captured using a snare, and exteriorized out of the right internal jugular vein, thereby establishing an arterial-venous circuit (Figure 1(b)). Next, the delivery sheath was advanced through the jugular vein into the LV, where the tip of the sheath was placed. After removal of the delivery sheath dilator and wire, the loaded flexible double-umbrella device was advanced through the delivery sheath, across the septal rupture, into the LV. The umbrella device was pushed partially out of its catheter sheath until release of the first umbrella. The delivery catheter was drawn back into the RV until the left-sided umbrella was positioned against the LV septum. Finally, the right-sided umbrella was released, covering the rupture from the right side. During this procedure, left ventriculography and echocardiographic control were performed to aid in guiding the device, visualizing the VSD, and assessing closure (Figure 1(c)).

### 2.5. Devices

Amplatzer™ VSD occluders (Figure 1(d)) depending on VSD size and morphology were used. The Amplatzer VSD occluders (AGA Medical Corporation, Plymouth, MN, USA) are self-expanding devices made of nitinol. Depending on the fabrication, the maximum left umbrella size is 32 mm for the recently released 24 mm Amplatzer muscular VSD occluder postinfarction (PI). The waist ranges from 7 mm for muscular VSD and 10 mm for muscular PI VSD occluders [11]. Polyester fabric inserts help close the VSD and provide a foundation for tissue growth over the occluder after deployment. Depending on the fabrication, the maximum occlusion size is 30 mm for the released muscular VSD occluders. All devices are secured to a delivery cable and inserted into a delivery sheath ranging from 8 to 12 French in size. The device size and type were chosen based on measurements of the VSD taken using left ventriculography and echocardiography.

### 2.6. Statistical Analysis

Quantitative variables were expressed as mean value ± standard deviation, and qualitative variables were expressed as total number and percentage. The independent two-sample* t*-test or one-way analysis of variance (ANOVA) with post hoc Student-Newman-Keuls test was used to assess the differences between multiple sets of data. Categorical variables were also compared using the chi-square or Fisher's exact test. Univariate and multivariate logistic regression analyses were used to identify independent predictors of in-hospital death. Death-free survival curves were constructed using Kaplan-Meier survival methods. Survival time was defined as the interval from admission or discharge to the time of death. Statistical significance was indicated when a two-sided* P* value was <0.05. All statistical analyses were performed using SPSS version 19.0 (SPSS Inc., Chicago, IL, USA).

## 3. Results

Among the 15 patients enrolled, 11 patients survived; the rest died during hospitalization as a result of severe lung infection and respiratory failure (1 case), LV rupture during device implantation (2 cases), or cardiac shock (1 case). The baseline clinical characteristics of the patients in the survival and death groups are given in Table 1. The patients who survived had significantly higher systolic blood pressure, diastolic blood pressure, and left ventricular ejection fraction at admission. The incidence of cardiac shock and heart failure of New York Heart Association (NYHA) class IV was significantly lower in the survival group than in the death group (*P* < 0.05).

### 3.1. Interventional VSD Closure Procedure

As shown in Table 2, patients who survived had significantly lower pulmonary/systemic flow ratio and shorter time from AMI to procedure compared with patients who died during hospitalization (*P* < 0.05). Additionally, there was a higher incidence of complete closure in patients who survived (72.72% versus 25.00%).

The procedural characteristics, complications, and vital status at follow-up are summarized in Table 3. Device placement was successful in 12 procedures (80%). The mean defect size was 14.20 ± 4.89 mm. Ventricular (*n* = 13) occluders were used, with a device/defect ratio of 1.56 ± 0.39. Two Amplatzer muscular VSD occluders were successfully placed in one patient (patient number 3).

### 3.2. Complications

As shown in Table 3, major immediate complications included residual shunting in 4 patients (26.67%), left ventricular rupture in 2 patients (13.33%), and device dislocation in 1 patient (6.67%). Two cardiogenic shock patients died during the operation as a result of LV rupture. In another patient, the VSD showed irregular shape, causing the device to dislocate into the RV. Therefore, the device had to be withdrawn. The patient presented with cardiogenic shock and died in the CCU 3 days postoperatively due to hemodynamic deterioration. In yet another patient, the VSD was too large, and a 24 mm VSD occluder was implanted. The patient complicated with a large residual shunt and died in the CCU 9 days postoperatively due to refractory heart failure.

### 3.3. Follow-Up

**Figure 2:**
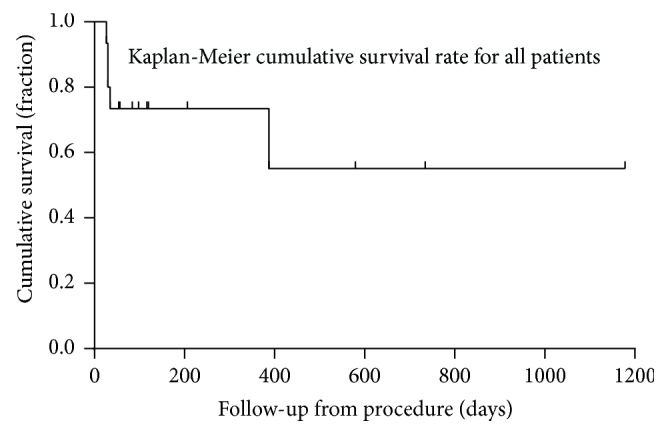
Kaplan-Meier cumulative survival curves indicating the estimated long-term survival for all patients.

**Figure 3:**
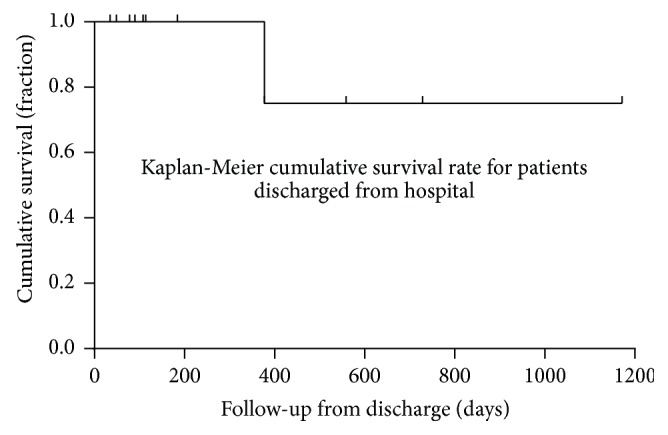
Kaplan-Meier cumulative survival curves indicating the estimated long-term survival for patients who survived until discharge from hospital.

A total of 4 patients died before discharge from the hospital, leading to an overall survival rate of 73.33%. The duration of the long-term follow-up for the patients who survived to hospital discharge was 249.67 ± 336.69 days. During this time, another patient died from cancer. No incidence of device or thrombus embolization was noted. Kaplan-Meier cumulative survival curves of the long-term survival for all patients and for patients who survived to discharge are displayed in Figures 2 and 3, respectively.

### 3.4. Risk Factors

In the univariate logistic regression analysis, cardiac shock (odds ratio, 30.00;* P* = 0.029) and heart failure of NYHA class IV (odds ratio, 30.00;* P* = 0.029) were correlated with in-hospital or 30-day mortality (Table 4).

## 4. Discussion

In the present study, the in-hospital and 30-day mortality for patients with successful device placement (12 cases) was 33.33%, which is well within the reported early mortality for surgical closure of postinfarction VSD (19–46%) [2, 4]. However, those who survived to hospital discharge had a good long-term outlook. Our experience suggests that percutaneous transcatheter closure of postinfarction VSD achieves a reasonable technical outcome, with a low rate of major complications. In previous studies, high 30-day mortality was usually attributable to multiorgan failure, poor systemic perfusion, and extensive comorbidity [12, 13]. In agreement with previous reports, we confirmed that cardiogenic shock at the time of clinical presentation and the hemodynamic burden imposed by the left-to-right shunt are important predictors of adverse outcomes [14].

Percutaneous transcatheter closure of postinfarction VSD has many advantages over surgical closure, including reduced trauma, shorter duration of the procedure, lower cost, higher rate of procedural success, and low in-hospital mortality rate. Therefore, this procedure is recommended for AMI patients with VSD in the following situations: patients with coronary artery disease referred for percutaneous coronary artery interventional therapy; elderly patients or patients who cannot undergo surgical treatment; patients with VSD rupture diameter of ≤15 mm; or abundant residual shunt after surgery [15, 16]. The majority of interventional postinfarction VSD procedures have been performed in such patients in the chronic or subacute phase [17]. Current guidelines recommend immediate surgical VSD closure irrespective of the patient's hemodynamic status, in order to avoid further hemodynamic deterioration [18, 19]. Thiele et al. [11] reported immediate complete VSD closure or initial hemodynamic stabilization in a prospective series of consecutive patients with VSD who underwent immediate primary transcatheter closure. At the 30-day follow-up, a total of 19 patients had died, leading to an overall mortality rate of 65%. It has been suggested that selection of “all-comers” with acute postinfarction VSD is probably responsible for the observed elevated mortality rate and that this represents the true mortality rate in VSD patients (i.e., without the selection bias). Meanwhile, Holzer et al. [20] described the outcomes of the procedure in 18 patients from different centers in the USA over a 3-year period. In the majority of patients, the procedure was attempted >2 weeks after infarction. Procedural success was 16 out of 18, and the 30-day mortality was 28%. Our experience is that transcatheter VSD closure after a delay of 10–14 days allows initial scarring of the surrounding tissue to occur and provides the opportunity to improve the NYHA class to II-III through medication.

Transcatheter interventional closure of postinfarction VSD is associated with a variable degree of residual shunts [21–23]. In the present study, although most of the residual shunts were trivial or small and passed through the fabric of the device, the patients did not tolerate persistent or large shunting after closure. Expert consensus suggests that if the shunt is not reduced by at least two-thirds, the patient is most unlikely to survive either to surgery or to discharge [24]. To reduce the risk of significant residual shunting, it is necessary to accurately predict the most appropriate size of the device. However, postinfarction VSD are typically serpiginous and often present multiple ruptures [25]. Multiple localized angiography and multipositional and multiplanar imaging are useful in this setting. We considered that assessing the size of the VSD by using a balloon was not necessary, because of the risk of enlarging the defect. The serpiginous and complex nature of the defects meant that the devices were sometimes unable to detect their real shape. In a previous study [22], a device 50% larger than the measured diameter of the VSD was used, in order to allow for enlargement of the VSD size due to lysis and ongoing necrosis of the tissues surrounding the VSD. Birnbaum et al. [1] suggested that the closure device itself may increase the size of the ventricular septal rupture. In our study, larger devices were selected, and a device/defect of ratio was 1.56 ± 0.39.

In our study, with an average interval from infarction to attempted occlusion of 11 days in survivors and 27 days in nonsurvivors, there was a significant trend in this direction. Mortality rates of 87~100% were reported for postinfarction VSD patients with cardiogenic shock in the multicentre, prospective GUSTO-I trial and the SHOCK-registry [11]. The results from these two studies probably represent most closely the true mortality rate of VSD patients without the selection bias that exists in most surgical or interventional based reports. In fact, the highest risk patients with ventricular dysfunction, shock, and unstable haemodynamic would have died before reaching the chronic/subacute phase of the disease process. Therefore, it would have to spend more time providing the opportunity to improve the general condition, cardiac function, and haemodynamic through medication in those highly unstable patients. Previous studies also demonstrate that, in selected patients, device closure is possible [20, 24]. However, in-hospital mortality is still high in those highest risk patients, even after apparently successful procedures.

## 5. Limitations

The present study is limited in several aspects. First, the size of the study population was small. Further investigations involving larger study populations are needed to confirm the present findings. Second, significant referral bias was present due to the retrospective and single-center design. Third, no direct comparison against traditional surgical closure was made. In the future, we plan to perform a randomized, multicentre, comparative study which is warranted in order to quantitatively assess the benefits of percutaneous closure over surgical closure.

## 6. Conclusions

Percutaneous closure of postinfarction VSD may be an effective alternative or adjunctive treatment to surgery. Despite achieving high procedural success rate, mortality was high for patients with shock. Nonetheless, although in-hospital mortality is high, patients who survived to discharge had excellent outcomes on long-term follow-up. Further developments of devices are needed in order to improve interventional outcomes. Finally, additional future multicentre studies are required in order to identify the safety and efficacy of percutaneous interventional closure for treating postinfarction VSD.

## Figures and Tables

**Table 1:** Demographic and clinical characteristics of the patients included in this study (*N* = 15). Patients who died during hospitalization were included in the death group, while those who survived were included in the survival group.

	Survival (*n* = 11)	Death (*n* = 4)	*P* value
Age, years	63.36 ± 6.36	62.25 ± 10.78	0.269
Men, *n* (%)	5 (45.45)	1 (25.00)	0.604
Body mass index, kg/m^2^	20.96 ± 2.74	20.72 ± 2.43	0.883
Current smoker, *n* (%)	3 (27.27)	3 (75.00)	0.235
Hypertension, *n* (%)	7 (63.64)	2 (50.00)	0.538
Hyperlipidemia, *n* (%)	10 (91.00)	3 (75.00)	0.476
Diabetes, *n* (%)	2 (18.18)	1 (25.00)	0.774
Admission SBP, mm Hg	110.72 ± 8.46	97.50 ± 15.20	**0.049**
Admission DBP, mm Hg	74.45 ± 6.17	61.50 ± 11.36	**0.013**
Heart rates, beats/min	87.27 ± 14.81	106.00 ± 20.46	0.071
Shock, *n* (%)	1 (9.10)	3 (75.00)	**0.033**
Infarct territory, *n* (%)			
Anterior	7 (63.64)	3 (75.00)	0.593
Inferior	4 (36.35)	1 (25.00)	0.593
Culprit lesion, *n* (%)			
LAD	7 (63.64)	3 (75.00)	0.593
LCX	2 (18.18)	1 (25.00)	0.774
RCA	1 (9.10)	1 (25.00)	0.476
NYHA classification, *n* (%)			
I	2 (18.18)	0 (0.00)	—
II	3 (27.27)	0 (0.00)	—
III	5 (45.45)	1 (25.00)	0.462
IV	1 (9.10)	3 (75.00)	**0.033**
LVEF, %	52.55 ± 6.27	44.75 ± 3.59	**0.037**
*β*-Receptor blocker, *n* (%)	6 (54.54)	2 (50.00)	0.662
ACEI/ARB, *n* (%)	7 (63.63)	2 (50.00)	0.538
Spironolactone, *n* (%)	7 (63.63)	3 (75.00)	0.593
Creatinine, *μ*mol/L	83.99 ± 19.64	76.72 ± 11.06	0.502

Mean values (standard deviation) and total number (percentage) are given for continuous and categorical variables, respectively. SBP: systolic blood pressure; DBP: diastolic blood pressure; LVEF: left ventricular ejection fraction; NYHA: New York Heart Association; ACEI: angiotensin-converting enzyme inhibitors; ARB: angiotensin receptor blocker.

**Table 2:** Procedural parameters.

	Survival (*n* = 11)	Death (*n* = 4)	*P* value
Time from AMI to procedure, days	11.09 ± 6.32	27.50 ± 1.73	**<0.001**

Vessels with CAD, *n* (%)			
1	3 (27. 27)	0 (0.00)	—
2	7 (63.63)	3 (75.00)	0.593
3	1 (9.10)	1 (25.00)	0.476

PCI performed	2	0	—
*Q*p/*Q*s	1.52 ± 0.25	2.12 ± 0.13	**<0.001**

Immediate reduction in shunting, *n* (%)			
No reduction	0	—	—
Partial reduction	3	0	—
Complete closure, *n* (%)	8 (72.72)	1 (25.00)	0.143

Mean values (standard deviation) and total number (percentage) are given for continuous and categorical variables, respectively. CAD: coronary artery disease; PCI: percutaneous coronary intervention; AMI: acute myocardial infarction; *Q*p/*Q*s: pulmonary/systemic flow ratio.

**Table 3:** Individual characteristics regarding patients, procedures, complications, and in-hospital follow-up.

Patient number	Age	Sex	VSD location	VSD size (mm)	Device (mm)	Implantation successful	Complication	Survival	Follow-up duration (days)
1	58	F	Muscular	15	VSD 24	+	−	+	35
2	59	F	Muscular	9	VSD 16	+	−	+	108
3	64	M	Muscular	10 & 12	VSD 14 & 16	+	Residual shunting	+	114
4	54	F	Muscular	16	−	−	LV rupture during device implantation	−	0
5	67	F	Muscular	11	VSD 20	+	−	+	378
6	57	M	Muscular	18	P.I. VSD 24	+	−	+	730
7	78	F	Muscular	18	VSD 24	+	Residual shunting	−	9
8	62	F	Apical	14	VSD 16	+	−	+	49
9	77	F	Muscular	9	VSD 14	+	−	+	185
10	58	M	Muscular	15	VSD 20	+	Residual shunting	+	90
11	57	F	Muscular	28	VSD 30	−	Dislocation of device into RV	−	3
12	58	M	Muscular	10	VSD 22	+	−	+	78
13	67	M	Muscular	10	VSD 24	+	Residual shunting	+	560
14	60	M	Apical	15	−	−	LV rupture during device implantation	−	0
15	70	F	Muscular	14	VSD 20	+	−	+	1174

RV: right ventricle; LV: left ventricle; M: male; F: female; VSD: ventricular septal defect; PI: postinfarction. Follow-up duration indicates days to last contact if alive or time to death.

**Table 4:** Univariate regression analysis for factors associated with in-hospital or 30-day mortality.

	OR (95% CI)	*P* value
Age	0.985 (0.817–1.123)	0.595
Male	0.125 (0.009–1.723)	0.12
Admission SBP	0.883 (0.768–1.015)	0.08
Admission DBP	0.819 (0.670–1.002)	0.052
NYHA class IV	30.000 (1.410–638.150)	**0.029**
Preop LVEF	0.674 (0.439–1.035)	0.071
Preop shock	30.000 (1.410–638.150)	**0.029**
Preop serum creatinine	0.973 (0.901–1.050)	0.457

CI: confidence interval; OR: odds ratio; SBP: systolic blood pressure; DBP: diastolic blood pressure; NYHA: New York Heart Association; LVEF: left ventricular ejection fraction.
